# Cerebral iron deposition in the Globus pallidus and Substantia nigra of aging dogs and cats presenting as bilateral hypointensity on T2w and iron-sensitive MRI sequences (SWI, T2*)

**DOI:** 10.3389/fvets.2026.1706134

**Published:** 2026-02-11

**Authors:** Carolin Fischer, Sebastian Schaub, Svenja Susanne Erika Körber, Monika Isabel Hoppe, Kathrin Büttner, Martin Jürgen Schmidt

**Affiliations:** 1 Department of Veterinary Clinical Sciences, Clinic for Small Animal Surgery, Justus-Liebig-University Giessen, Giessen, Germany; 2 Department of Veterinary Pathology, Justus-Liebig-University Giessen, Giessen, Germany; 3 Department for Biomathematics and Data Processing, Justus-Liebig-University Giessen, Giessen, Germany; 4 Department of Veterinary Clinical Sciences, Clinic for Small Animals, Neurosurgery, Neuroradiology and Clinical Neurology, Justus-Liebig-University Giessen, Giessen, Germany

**Keywords:** accumulation, brain, iron, MRI, neurodegenerative

## Abstract

**Introduction:**

With aging, dysregulation of brain iron homeostasis can lead to cerebral iron accumulation, a process associated with the pathogenesis of neurodegenerative diseases and recognized as an imaging marker in humans. Similarly, MRI-detected cerebral iron accumulation has been reported in aging beagles, suggesting parallels with human findings.

**Methods:**

Single-center, observational study that retrospectively and prospectively evaluated 236 client-owned animals (198 dogs, 38 cats) undergoing brain MRI between 2014 and 2024. Patients represented various breeds and ages; scans were performed at different field strengths, 1-3 Tesla (T), with each animal examined once at a single field strength. MRIs were evaluated for bilateral hypointensities in T2-weighted (T2w) and iron-sensitive imaging (SWI, T2*) in the Globus pallidus and Substantia nigra. In selected cases, iron deposition was validated via post-mortem iron-sensitive Perl’s staining of fixed brain tissues.

**Results:**

Logistic regression analysis identified age as a significant predictor of improved visibility (OR = 1.21, *p* < 0.0001). Compared with 1T, 1.5T increased the odds of visibility by 2.78-fold (*p* = 0.0366), and 3T by 4.82-fold (*p* < 0.001). Species-specific analysis showed that, in cats, field strength had no significant effect (*p* = 0.1575), whereas age remained a significant predictor (*p* = 0.0192). Iron-sensitive sequences (SWI, T2*) provided superior visibility of iron deposits compared to T2w, particularly in dogs. No significant difference in hypointensity detection was observed between the two brain regions.

**Discussion:**

In summary, age and MRI field strength significantly affect the visibility of cerebral iron deposits in dogs, whereas feline results require further investigation due to the limited sample size. SWI and T2* enhance iron detection compared to T2w, particularly in canines.

## 1 Introduction

Magnetic Resonance Imaging (MRI) has become an essential tool in veterinary neurology, providing non-invasive, high-resolution imaging of brain structure in humans and animals. One of its evolving applications is the detection of cerebral iron (1) deposits, which are recognized as significant in the context of physiological aging but also neurodegenerative diseases in humans (2).

Iron is essential for normal brain function (3). Of all transition metals in the brain that are critical due to their chemical versatility, iron is the most abundant and present at higher concentrations than other transition metals (copper, zinc, or manganese) (4). Iron supports the high metabolic demands of neuronal tissue by enabling oxygen transport, mitochondrial function, neurotransmitter synthesis and myelination due to its ability to act as an electron donor and acceptor (2, 5–7). A deficiency of iron during brain development can result in irreversible developmental impairments (8), whereas excessive accumulation of iron can cause neurotoxic effects that disrupt normal brain function (9, 10). Therefore, iron concentrations are carefully regulated by cellular transport systems and iron-binding proteins, such as ferritin, to maintain a balance between adequate supply and potential toxicity (2).

Most of the body’s iron is stored as heme iron in red blood cells (bound to hemoglobin). Non-heme iron circulates primarily bound to transferrin, delivering iron to peripheral tissues (11) or is stored as ferritin or hemosiderin within macrophages or parenchymal cells in organs involved in iron storage (e.g., liver, spleen, bone marrow), erythrocyte breakdown, or at sites of bleeding (12). Entry of iron into the central nervous system (CNS) is closely regulated by the blood–brain barrier (BBB). Iron bound to transferrin crosses the BBB via receptor-mediated endocytosis through the endothelial cells that line cerebral capillaries (13–15). Once iron has entered the brain, it exists in two forms: ferric iron (Fe^3+^), typically bound to transferrin extracellularly and to ferritin intracellularly (the main iron-storage protein); and ferrous iron (Fe^2+^), existing in intra- and extracellular spaces as free iron, which contributes to redox reactions and cellular metabolism (16).

Evidence from human and rodent studies shows that cerebral iron accumulation begins early in life, with deep gray matter structures such as the Globus pallidus (GP) and Substantia nigra (SN) among the first regions affected, and continues progressively through adulthood (4, 17, 18). The GP, a subcortical grey matter nucleus, is located medial to the putamen and lateroventral to the internal capsule. The SN lies dorsomedial to the crus cerebri (19). Normally, protective mechanisms, including iron-binding proteins (such as ferritin), maintain iron homeostasis and prevent toxicity (3, 5, 10). With aging, however, this regulatory balance can be compromised, and gradual iron accumulation in the SN, GP, red nucleus, and putamen can shift iron toward its reactive ferrous form, thereby contributing to oxidative stress via mechanisms such as the Fenton reaction (2, 7, 10, 20–22). This generates harmful reactive oxygen species (ROS), particularly the highly cytotoxic hydroxyl radical (⋅OH), which can cause redox imbalance, mitochondrial dysfunction, lipid peroxidation, ferroptosis, DNA damage, and protein oxidation (7). These mechanisms are considered hallmarks of neuronal injury and age-related neurodegenerative decline (5, 20, 23). Zecca et al. (4) reported that age-dependent increases in iron content within the SN and GP of rats correlated with elevated oxidative stress markers and neuronal vulnerability. Therefore, brain iron accumulation has been implicated in the pathogenesis of several human neurodegenerative diseases, including Parkinson’s (4, 9, 24–26) and Alzheimer’s disease (27–29), as well as a group of disorders categorized as Neurodegeneration with Brain Iron Accumulation (NBIA) (30).

Dogs, like humans, undergo age-related cognitive changes (31, 32), making them relevant models for studying brain aging and neurodegeneration (26, 33–38). In an experimental study of 19 aging beagle dogs, iron accumulation was visualized as T2w hypointensities in the GP and SN using a non-commercial, pre-clinical 4.7 Tesla (T) high-field MRI scanner and has been associated with aging, paralleling findings observed in human medicine (33). In T2w sequences, iron induces signal loss by generating local magnetic field inhomogeneities, which shorten T2 relaxation times and result in a hypointense signal intensity (39, 40). Advances in MRI techniques, especially T2*-weighted (T2*) gradient-echo (GRE) and susceptibility-weighted (SWI) sequences, have enhanced the *in vivo* detection of cerebral iron (41–46). T2* imaging is inherently sensitive to magnetic susceptibility effects from iron deposition, which create local field inhomogeneities and faster spin–spin relaxation (42, 47), while SWI further increases iron detection by incorporating both magnitude and phase data, an effect that is particularly pronounced at higher field strengths (41–46, 48, 49). Consequently, these MRI sequences are considered iron-sensitive (45, 46) and may reveal iron accumulation that is not yet detectable on conventional T2w imaging, thereby improving diagnostic sensitivity for iron.

This study examines the visibility of bilateral hypointensities on T2w and iron-sensitive MR sequences (SWI and T2*) in the GP and SN in a large cohort of dogs and cats, using clinically available MRI systems with standard high-field strengths (1-3 T). This approach explores the potential for non-invasive detection of cerebral iron accumulation without reliance on preclinical or ultra-high-field research scanners. We aimed to assess the frequency and detectability of these imaging features and investigated their association with age. We hypothesized that (1) bilateral T2w and SWI/T2* hypointensities representing iron deposition in the GP and SN would be more frequent in older animals, and (2) these changes would be more readily detectable with iron-sensitive sequences compared to conventional T2w sequences.

Detecting cerebral iron accumulation with MRI represents a promising, non-invasive approach to studying neuronal aging in companion animals. While iron-sensitive imaging has been extensively explored in human neurodegenerative research, systematic evaluations in veterinary patients, particularly across species, MRI sequences, and field strengths, remain limited. The present study aims to establish clinically applicable imaging markers of brain aging and to define methodological factors that influence their detectability. The ability to identify cerebral iron deposits on standard clinical MR scanners could enable earlier diagnosis and improve understanding of neurodegenerative diseases. When combined with cognitive function tests, this approach may also help monitor and track the progression of age-related decline.

## 2 Materials and methods

### 2.1 Animals

In this single-center observational study, both retrospective and prospective approaches were used. Specifically, medical records of dogs and cats that underwent brain MRI from January 2014 to January 2024 were reviewed for potential inclusion. MRI scans were performed for diverse purposes not associated with the focus of this study. In all cases, clients provided informed consent for the use of patient data and for institutional animal care at hospital admission; therefore, formal ethics committee approval was not required. Patients were suitable for inclusion only if their brain MRI protocol included, at a minimum, T2w (sagittal, dorsal, and transverse plane); transverse iron-sensitive sequences (SWI or T2*); transverse Fluid-attenuated inversion recovery (FLAIR); as well as T1-weighted (T1w) images acquired before and, when available, after contrast administration. In addition, all dogs and cats were required to have structurally normal brains and to undergo cerebrospinal fluid (CSF) analysis within 24 h of MRI, demonstrating normal cerebrospinal fluid physiology. Animals were excluded if MRI sequences were incomplete or of insufficient diagnostic quality, if intracranial pathologies were present, such as neoplastic, hemorrhagic, or inflammatory diseases, or if CSF abnormalities were detected. Following these criteria, eligible dogs and cats were enrolled consecutively, and of the more than 3,000 brain MRI scans reviewed, 236 client-owned animals (198 dogs and 38 cats) were included. The patients were of varying age (juvenile to aged) when MRI was performed, numerous breeds, and diverse weight classes (<10 kg, 10–30 kg, <30 kg). If a patient met the inclusion criteria more than once, only data from the first examination were used. The patient data collected from the medical records included signalment, including clinical history, presenting complaints, age at the time of MRI, weight, and CSF analysis results. Upon initial presentation, all patients underwent neurological examination by either a board-certified veterinary neurologist or a veterinary neurology resident in training under supervision; neurologic status was recorded in the patient’s medical record. After anamnesis, physical and neurologic examination, MRI studies were conducted due to one or more of the following symptoms: epileptic seizures, behavioral changes like aggression, vestibular syndrome, facial nerve paralysis, or blindness. Following physiological brain MRI scans and normal CSF results, the final clinical diagnosis was other than structural or inflammatory intracranial diseases: e.g., primary epilepsy, idiopathic facial nerve paralysis, otitis media. However, in some cases, a definitive clinical diagnosis could not be established.

### 2.2 MR imaging

All MR imaging examinations were performed at the diagnostic imaging section of the clinic for small-animal surgery and neurology, Justus-Liebig-University Giessen, Germany. Over the 10-year study period, several changes in imaging equipment occurred. Examinations performed between 2014 and 2016 were acquired using a 1T field-strength magnet (Philips Gyroscan Intera), whereas studies conducted between 2016 and 2021 were obtained using a 3T scanner (MAGNETOM Verio 3 Tesla, Siemens Healthcare GmbH). Examinations performed since September 2022 were acquired using a 1.5T scanner (MAGNETOM Avanto Tim-System Siemens Healthcare GmbH). Images were acquired using a loop coil for patients weighing <5 kg and a medium flex coil for patients weighing >5 kg. In all cases, a conventional clinical MRI protocol was performed, including at least T2w images in dorsal, transverse, and sagittal planes, transverse FLAIR, transverse SWI or T2*, and a T1w-3D sequence acquired before and after intravenous administration of a gadolinium-chelated contrast agent (Omniscan®).

For the purpose of this study, susceptibility-sensitive MRI techniques (SWI and T2*-weighted GRE imaging) used to detect iron-related signal loss are collectively referred to as iron-sensitive sequences. Iron-sensitive imaging was performed using either a T2* sequence or SWI, depending on scanner availability and time period. At 1T, a T2* sequence was used in all patients. On the 3T system, T2* imaging was performed during the initial period and was later replaced by SWI after October 2018. On the 1.5T system, SWI was used exclusively. No patient was examined with both T2* and SWI imaging.

All patients were examined under general anesthesia, using individually tailored anesthetic protocols. Following endotracheal intubation, anesthesia was maintained with isoflurane (1–3 Vol %; CP Pharma GmbH, Germany) in oxygen and room air. During general anesthesia, the monitored parameters included heart rate, oxygen saturation, and end-tidal carbon dioxide. All patients were positioned in standardized dorsal recumbency, with the forelimbs flexed and secured over the thorax.

### 2.3 Image analysis

**Figure 1:**
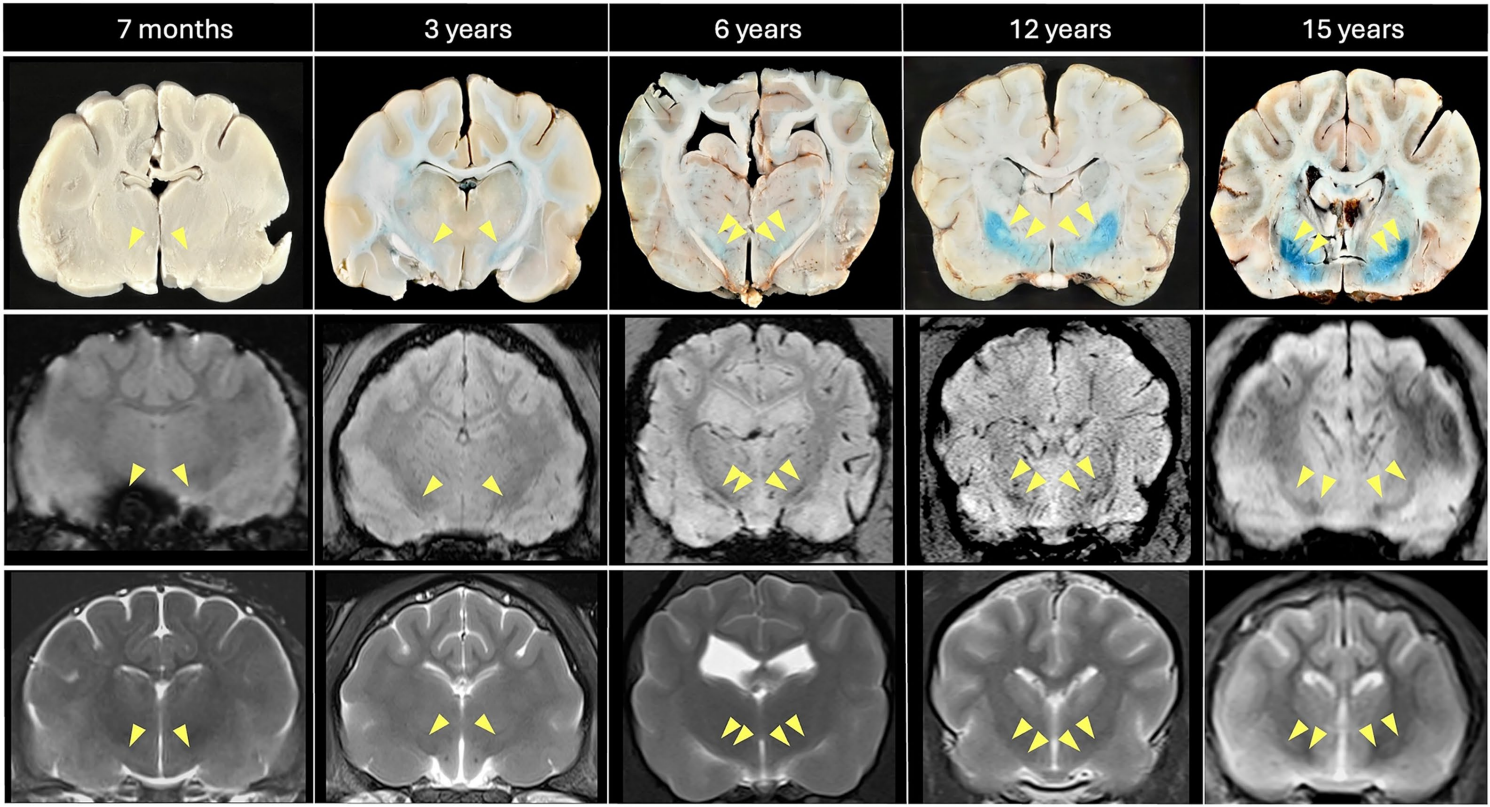
Comparison of MRI (SWI middle row: slice thickness 1.2 mm; T2w lower row; slice thickness 2–2.5 mm) with iron-sensitive Perls’-stained, formalin-fixed brain sections at the level of the Globus pallidus, indicated by yellow arrowheads (left to right: feline 7 months, feline 3 years, canine 6 years, canine 12 years, feline 15 years). Age-related iron accumulation, confirmed by intensified Perls’ staining, is reflected by progressively more conspicuous bilateral hypointensities (SWI > T2w) with increasing age. Note the marked susceptibility artifact in the right ventral brain (SWI, 7-month-old cat) caused by a post-mortem air-tissue interface.

**Figure 2:**
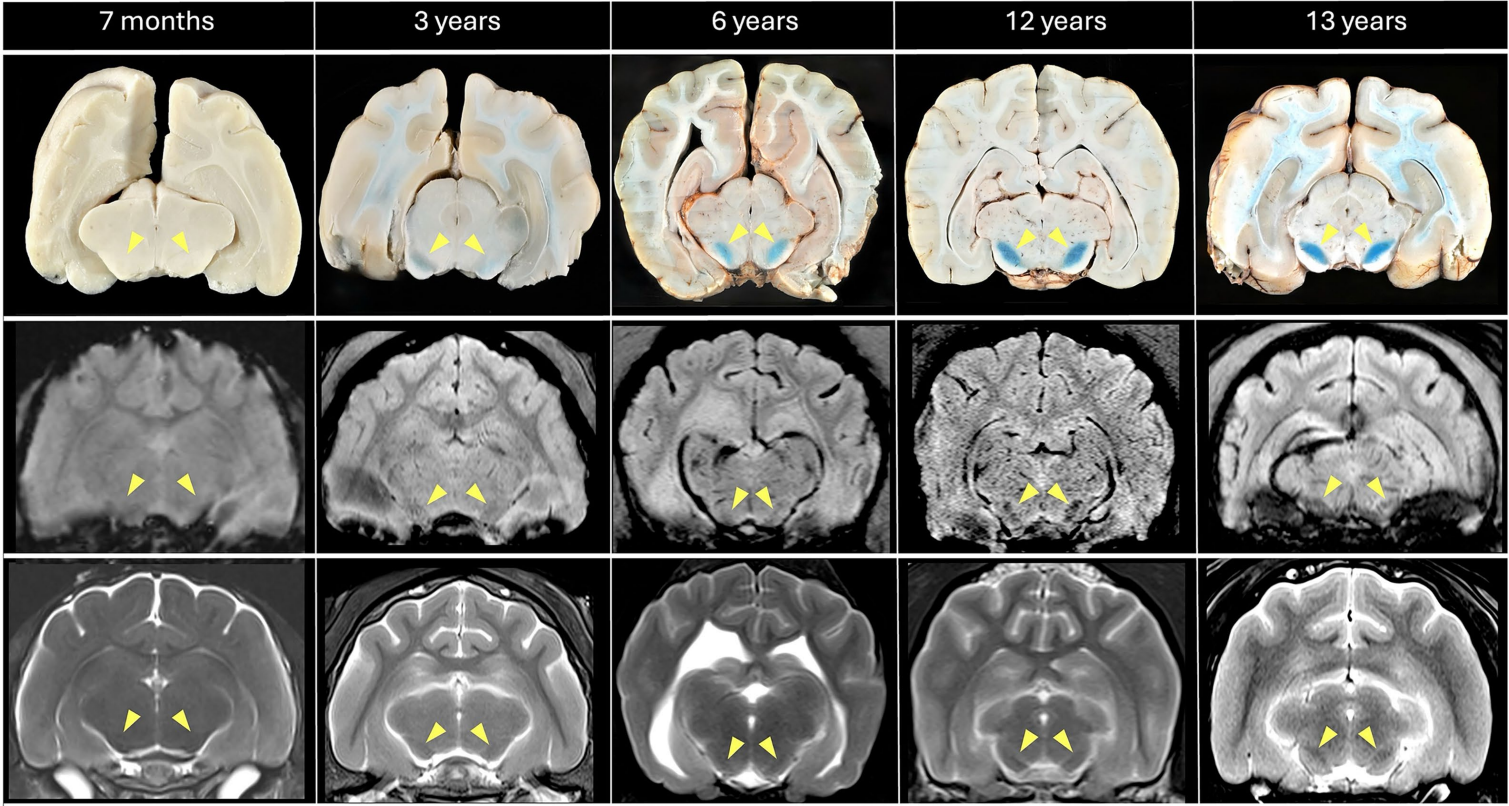
Comparison of MRI (SWI middle row: slice thickness 1.2 mm; T2w lower row; slice thickness 2–2.5 mm) with iron-sensitive Perls’-stained formalin-fixed brain sections at the level of the Substantia nigra, indicated by yellow arrowheads (left to right: feline 7 months, feline 3 years, canine 6 years, canine 12 years, feline 13 years). Age-related iron accumulation, confirmed by intensified Perls’ staining, is reflected by progressively more conspicuous bilateral hypointensities (SWI > T2w) with increasing age. Note the mild bilateral susceptibility artifact in the ventral brain (SWI, particularly in the 3- and 13-year-old cats), attributed to air-tissue interfaces and partial volume effects from adjacent pneumatized tympanic bulla.

MR images were accessed from the Picture Archiving and Communication System (PACS) and examined using an open-source imaging viewer (HorosTM, The Horos Project, Purvis, Annapolis, Maryland, USA). Importantly, the observers were blinded to medical record findings during MR image evaluation. Images were reviewed across all available sequences and evaluated for bilateral symmetric T2w- and SWI/T2*-hypointense areas within predefined regions of interest (ROI). Specifically, ROIs were defined at the level of the GP and SN using a canine (50) and feline (51) histologic brain atlas. The presence of hypointensities within these ROIs was evaluated in transverse T2w and iron-sensitive (SWI, T2*) images, where it appeared as a focal ovoid to crescent-shaped, mostly bilateral symmetric area coursing dorsolaterally and intermittently across multiple consecutive slices (Figures 1, 2). The presence, absence, and degree of visibility of these hypointense areas within the predefined regions were recorded for each sequence. Visibility and signal intensity were graded subjectively on a scale from 0 to 2, where 0 indicated absence, 1 indicated faintness, and 2 indicated strong visibility. To enhance the reliability of image analysis, an intraobserver assessment was performed to evaluate the consistency of repeated evaluations. Sixty transverse T2w and iron-sensitive sequences (including SWI, *n* = 31, and T2*, *n* = 29) were randomly selected from the study population (20 from each MRI field-strength group, including both canine and feline patients) and independently reviewed twice by the same observer. This double review tested the reproducibility of the previously applied grading system.

### 2.4 Postmortem Perl’s staining to validate iron deposition

To validate the presence of cerebral iron, Perls’ Prussian blue staining was performed on postmortem brain tissue from a selected subset (*n* = 10) of canine (*n* = 5) and feline (*n* = 5) brains of diverse ages that exhibited various degrees of T2w- and iron-sensitive hypointensities on MRI. Following euthanasia for reasons unrelated to this study, within 24 h post- mortem, the cranium was opened dorsally by removal of the frontal, parietal and occipital bones, and the remaining skull and brain were placed in 10% neutral buffered formalin for fixation. After at least 10 days, the fixated brain was carefully extracted and thoroughly rinsed with distilled water. Subsequently, neurological tissue was sectioned into 4 mm slices in the regions of interest and, including a positive iron control on a microscopic slide, incubated at room temperature for 30 min in a freshly prepared 1:1 mixture of 2% potassium ferrocyanide (K_4_[Fe(CN)_6_]) and 2% hydrochloric acid (HCl) (Perl’s Prussian blue staining). This reaction produces an insoluble blue pigment (ferric ferrocyanide) in regions of the brain containing iron. Following incubation, slides were rinsed in distilled water; to prevent metal contamination, plastic-based forceps were used for handling of the brain slices. As a result, blue-stained iron deposits were identified in the GP and SN, corresponding to the previously detected MRI hypointensities (Figures 1, 2). In the youngest subject (a 7-month-old cat), iron levels were below the detection threshold of Perl’s stain.

### 2.5 Statistical analysis

Statistical analyses were conducted using SAS® software (SAS Institute Inc., 2013. Base SAS ® 9.4 Procedures Guide: Statistical Procedures 2nd ed.). All analyses were performed by the head of the university’s Biomathematics and Data Processing Department. Intraobserver agreement was conducted by the same observer evaluating a subset of 60 MRI sequences twice. Agreement was quantified using percent agreement, Spearman’s rank correlation coefficient, and the Kappa statistic. These measures were calculated for visibility scores in the GP and SN, separately for T2w and iron-sensitive sequences, with SWI and T2* analyzed collectively as a single category. Results were categorized by species (dog and cat) and presented with 95% confidence intervals. To evaluate whether visibility scores were associated with patient age or field-strength (1T, 1.5T, or 3T), cumulative logistic regression models were applied, including species-combined and species-specific models. To assess categorial differences between visibility scores by sequence type (T2w vs. iron-sensitive), Tesla, species, age, and brain region, chi-square tests and Fisher’s exact tests were used depending on the sample size. To compare visibility scores between T2w and iron-sensitive sequences within the same brain region, Bowker’s symmetry test for paired ordinal data was applied. If a significant asymmetry was detected, *post hoc* pairwise comparisons were performed with Bonferroni correction. Direct comparisons of the scores between the GP and SN across all sequences were performed using Bowker’s test of symmetry, applied separately for the whole dataset and for canine and feline subgroups. This test assessed whether visibility ratings differed significantly between the two anatomical regions. Significance was defined as *p* < 0.05 for all analyses.

## 3 Results

**Figure 3:**
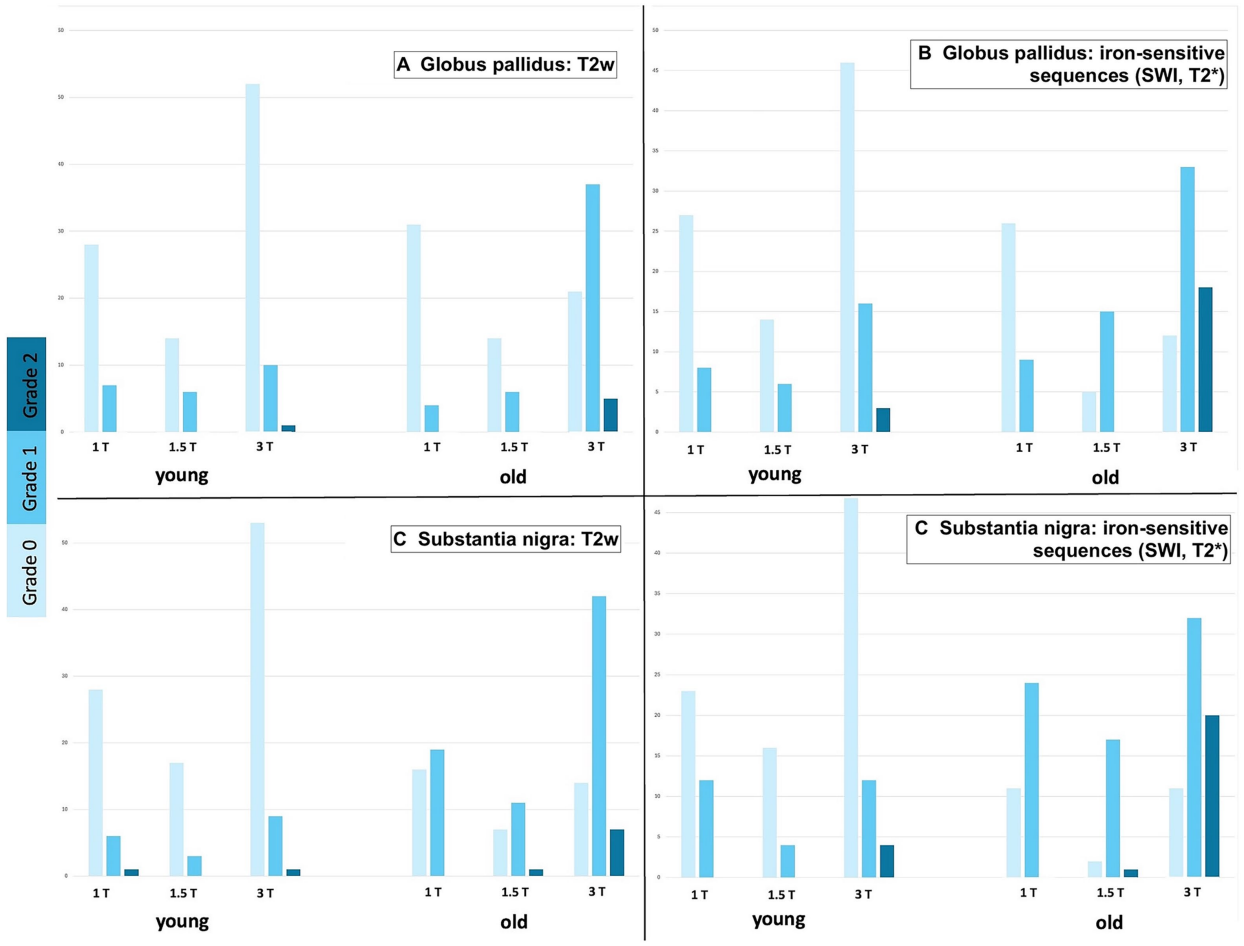
Clustered column charts showing grade distributions (0: light blue, 1: medium blue, 2: dark blue) across the entire study pool. For each region of interest [Globus pallidus **(A,B)** and Substantia nigra **(C,D)**] across MRI sequences [T2w **(A,C)** and iron-sensitive **(B,D)**], stratified by age group and magnetic field strength. For each field strength (1T, 1.5T, 3T), the left columns represent younger patients (<7 years), and the right columns represent older patients (≥7 years). Iron-sensitive sequences consistently yielded higher visibility scores than T2w in both regions, across all age groups and field strengths. Note that in older age groups, higher grades (1 and 2) were significantly more frequent, reflecting the age-related nature of iron accumulation.

**Figure 4:**
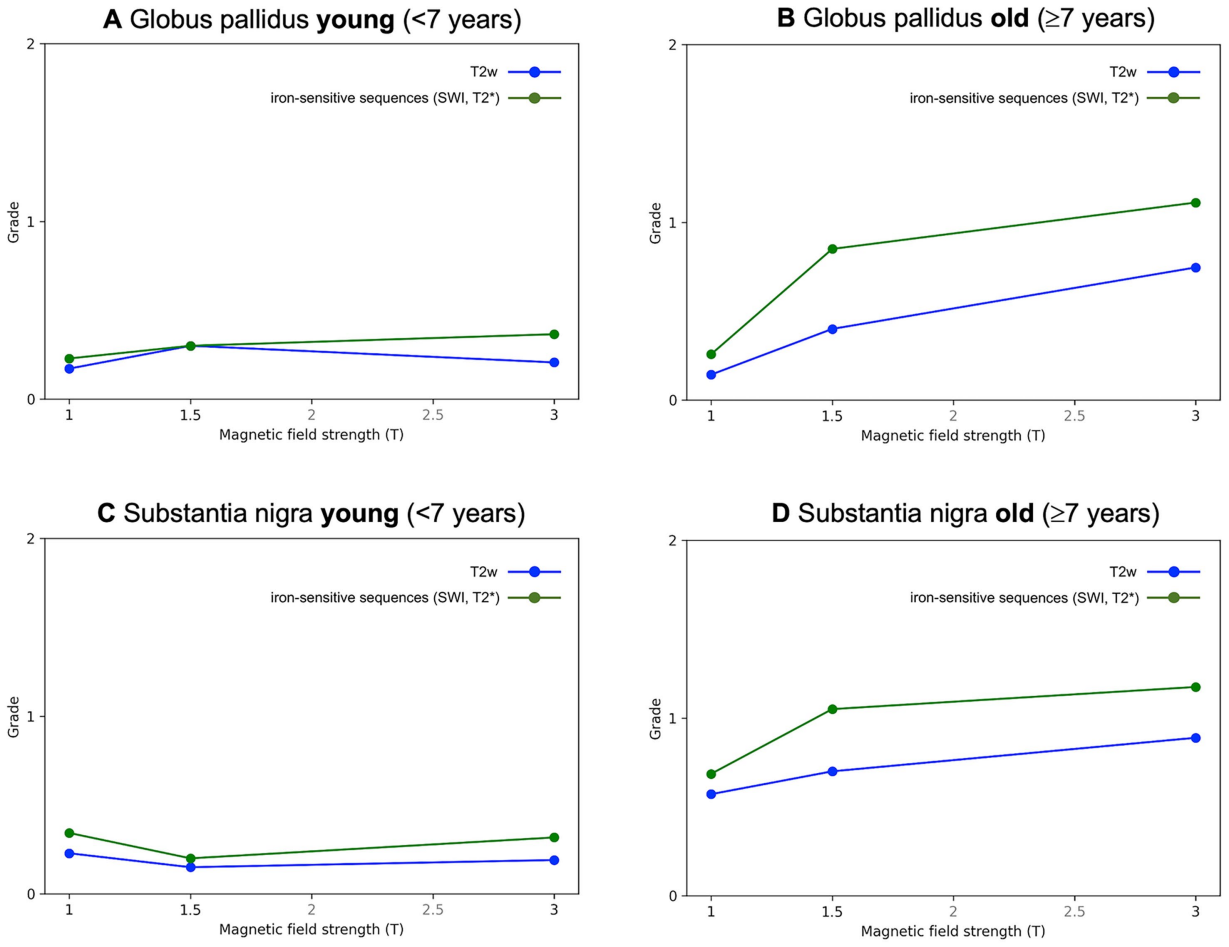
Linear trends of hypointensity grades (0–2) across magnetic field strengths [1T, 1.5T, 3T]. Separate plots are shown for young [<7 years; **(A,C)**] and older [≥ 7 years; **(B,D)**] animals, stratified by brain region [Globus pallidus **(A,B)**, Substantia nigra **(B,D)**] and sequence type (T2w, blue; iron-sensitive, green). The figure highlights the age-related increase in iron deposition and shows higher hypointensity grades on iron-sensitive sequences than on T2w, particularly at higher magnetic field strengths.

The study population comprised 236 animals; an overview of species distribution, age groups, MRI field strengths, and employed sequences is provided in Table 1. Intraobserver agreement was generally high. For T2w grading of the GP, the overall percent agreement was 87%, with a Spearman’s correlation coefficient (rs) of 0.72 and a Kappa value of 0.70. Agreement was particularly high in cats (94% agreement, rs = 0.86, *κ* = 0.85), whereas in dogs it was slightly lower but still acceptable (83% agreement, rs = 0.70, *κ* = 0.65). For the SN, agreement was even stronger overall. The T2w assessment showed 93.3% agreement (rs = 0.87, *κ* = 0.87), with nearly perfect (97.6%) agreement in dogs (rs = 0.66, *κ* = 0.95). Agreement in cats remained acceptable (83.3%, rs = 0.66, *κ* = 0.66). Similar levels of agreement were observed for the combined T2w and iron-sensitive sequence scores. Logistic regression revealed that both age and field strength were significant predictors of visibility. Older animals were more likely to show increased visibility (OR = 1.21, 95% CI: 1.11–1.31, *p* < 0.0001). Visibility also improved with stronger magnetic fields: 1.5 Tesla was associated with a 2.68-fold increase in the odds of increased visibility (*p* = 0.0366), and 3 Tesla with a 4.82-fold increase (*p* < 0.0001). In feline models, age remained a significant factor (OR = 1.257, *p* = 0.0192), but the effect of field strength was not statistically significant (*p* = 0.1575), possibly due to the smaller sample size. Comparison of T2w and iron-sensitive sequences showed that iron-sensitive imaging tended to yield higher visibility scores in both the GP and SN (Figures 3, 4). Bowker’s test indicated significant differences in visibility between the two sequences (*p* < 0.0001), particularly in dogs. *Post hoc* testing confirmed that in iron-sensitive imaging, cases were more frequently classified as grades 1 and 2 than in T2w (*p* = 0.0001 and *p* = 0.0008, respectively). In cats, the trend was similar but did not reach statistical significance (*p* = 0.055). Finally, a direct comparison of visibility scores between the GP and SN was performed to assess regional differences. Bowker’s test revealed no statistically significant differences in visibility distributions between these two anatomical regions in the whole dataset (*p* = 0.0827), as well as for each subgroup (canine *p* = 0.2642; feline *p* = 0.2998).

**Table 1:** Study population characteristics and MRI equipment/sequences used.

Variable		Total (*n* = 236)		Dogs (*n* = 198, 84%)	Cats (*n* = 38, 16%)
Age at MRI		1–190 months (0.1–15.8 years)			
Age distribution					
< 7 years		118 (50%)		100	18
≥ 7 years		118 (50%)		98	20
MRI field strengths					
3T		126 (53%)		105	21
1.5T		40 (17%)		32	8
1T		70 (30%)		61	9
MRI sequences					
1T	T2w	236 (100%)		198	38
1.5T					
3T					
*Iron-sensitive sequences (total)*					
SWI		121 (51%)		97	24
T2*		115 (49%)		101	14
*Iron-sensitive sequences (in various field strengths)*					
1T	T2*	70 (100%)		61	9
1.5T	SWI	40 (100%)		32	8
3T	T2*	45 (36%)	23 (51%) < 7y	40	5
			22 (49%) ≥ 7y		
	SWI	81 (64%)	40 (49%) < 7y	65	16
			41 (51%) ≥ 7y		

*Belongs to the MRI sequence (T2*) sequence.

## 4 Discussion

This study supports the concept of cerebral iron buildup in aging dogs and cats and shows that clinically available high-field MRI systems (1-3T) are suitable for its non-invasive detection. In line with our first hypothesis, bilateral T2w and SWI/T2* hypointensities indicating iron accumulation were significantly more common in older animals, supporting an age-related increase in cerebral iron deposition. Additionally, consistent with our second hypothesis, these signal changes were more easily detected in iron-sensitive sequences than in traditional T2w. Despite differences in sample size, no significant interspecies differences were observed in the anatomical distribution or prevalence of MRI-visible iron deposition, suggesting a common biological mechanism across species.

### 4.1 Age-related changes and validation by Perls’ staining

We found a significant association between age and the presence of MRI-visible hypointensities in the GP and SN. Logistic regression analysis showed that age was significantly associated with increased MRI visibility of iron, with each additional year of age increasing the odds of detecting iron deposition by 21% across the study population. In the species-specific analysis, this association was also observed in cats; however, the limited sample size reduced the statistical power of the analysis. These findings are aligned with earlier veterinary studies and consistent with the literature on age-associated iron accumulation in humans and animal models (25, 33, 52, 53).

Post-mortem Perl’s blue staining supports the interpretation that age-associated hypointensities observed on T2w and iron-sensitive MRI are consistent with progressive regional iron accumulation in dogs and cats, consistent with observations in aging human and rodent brains (4, 17, 18). The preferential iron accumulation observed in the GP and SN in our study likely arises from the same fundamental age-related processes of iron handling and oxidative metabolism across mammalian species due to the high baseline iron content, dense mitochondrial activity, and vulnerability to oxidative metabolism of these brain regions, which may promote iron sequestration with advancing age (4, 54, 55).

The closer spatial and morphological correspondence between blue-stained iron deposits and hypointensities on iron-sensitive imaging further emphasizes the superior sensitivity of susceptibility-based imaging to paramagnetic compounds that disturb the local magnetic field during MRI (41–46). Because of the predominantly retrospective design and the variability of our study population (ranging from small juvenile cats to large adult dogs) and imaging equipment, we could not maintain consistent imaging protocols. As a result, small differences in slice thickness between MRI sequences and post-mortem brain sections, leading to minor spatial discrepancies in regional correspondence, could not be completely avoided. In the 7-month-old kitten, no macroscopic iron deposition was detected using Perls’ staining. This may reflect the physiological reality that cerebral iron levels in very young subjects remain below the detection threshold of macroscopically visible Prussian blue staining. Age-dependent thresholds for detectable non-heme iron have been described in other species, including humans and rodents, suggesting that significant iron accumulation in deep grey matter nuclei such as the GP and SN may not occur until later developmental stages (40, 54). Alternatively, the absence of Perl’s staining in this case may have been due to technical factors, particularly prolonged formalin fixation, which may have reduced staining sensitivity. Extended exposure to formalin has been shown to cause iron leaching from tissues, leading to diminished staining intensity and potential false-negative results (56, 57). Due to the limited availability of age-matched, neurologically normal brains in this cohort, these interpretations remain speculative. Importantly, a clear timeline for the onset of detectable brain iron deposition in companion animals has not yet been established in the literature and warrants further investigation.

### 4.2 MRI sequences and field strength

The increased conspicuity of iron-sensitive imaging compared with conventional T2w imaging for detecting age-related cerebral iron deposition is consistent with the physical principles of susceptibility-based contrast. In the present study, iron-sensitive imaging comprised susceptibility-based techniques, including SWI and T2*, which were analyzed collectively.

On conventional T2w imaging, signal contrast is generated using spin-echo refocusing, which largely compensates for static magnetic field inhomogeneities. As a result, iron-related signal loss becomes apparent mainly at longer echo times and higher tissue iron concentration, where accelerated spin–spin dephasing leads to faster T2 decay and reduced signal intensity (52). Previous studies suggest that regional iron levels of approximately 10-15 mg/100 mg tissue are required for detectable T2 shortening at 1.5 Tesla (52). Consequently, conventional T2w sequences may be less sensitive to mild or early-stage iron deposits, particularly at lower field strengths.

In contrast, T2* uses a gradient-echo acquisition without refocusing pulses, allowing susceptibility-induced microscopic magnetic field variations caused by paramagnetic substances such as iron to accumulate even at shorter echo times, thereby increasing sensitivity to iron deposition (47).

SWI further enhances this effect by combining magnitude and phase information to accentuate subtle susceptibility effects, particularly at higher magnetic field strengths (39, 43, 48, 49). Although SWI is generally considered more sensitive than conventional T2* imaging due to its phase-based contrast enhancement, both techniques rely on the same fundamental susceptibility mechanisms can depict iron-related signal loss, supporting their collective evaluation in this largely retrospective study.

Accordingly, the greater detectability of iron-associated hypointensities observed on iron-sensitive imaging in our cohort is therefore in line with findings from human neuroimaging studies demonstrating superior sensitivity of susceptibility-based techniques compared with conventional T2w imaging (39, 43, 44, 48, 49, 58, 59). A direct within-patient comparison of SWI and T2* was not possible due to scanner-related protocol changes during the study period, as no patient underwent both techniques.

While R2* relaxometry (60–62) and quantitative susceptibility mapping (QSM) (25, 62, 63) represent even more powerful iron-sensitive MRI modalities, they were beyond the scope of this study, as they require standardized acquisition protocols and dedicated post-processing. These methods offer a quantitative assessment of iron load. R2* exploits the paramagnetic nature of iron to induce magnetic field inhomogeneities (64). Whereas QSM, reconstructed from raw (unfiltered) gradient-echo phase data, provides a quantitative measure of local magnetic susceptibility and correlates more directly with tissue iron content (25, 64–66). In our study, intraobserver agreement for image interpretation was high (87% overall, 94% in cats), demonstrating the reliability of the applied scoring system. However, the slight deficit relative to ideal intra-observer benchmarks (90–95%) suggests that additional standardization, such as QSM, may further improve reproducibility. Nonetheless, the consistent presence of hypointensities on SWI and T2* in regions typically associated with iron accumulation, such as the GP and SN, support the clinical utility of these widely available sequences in routine MRI protocols.

Despite its high conspicuity in iron detection compared to T2w, iron-sensitive imaging is inherently more susceptible to noise and artifacts (43, 48), particularly in ventral brain regions at the level of the SN. Susceptibility effects arising from adjacent air-filled structures, such as the tympanic bullae and, rostrally, the frontal sinuses, may degrade image quality (43, 48) and obscure anatomical detail (Figure 2). Additionally, susceptibility-based techniques are not specific to iron, as signal loss may also result from diamagnetic substances, such as calcium (43). Although phase imaging can theoretically differentiate paramagnetic and diamagnetic materials depending on the MRI coordinate system (left- vs. right-handed) (67, 68), access to phase data is often limited in routine clinical practice. Nevertheless, the strong concordance between iron-sensitive staining and imaging in the examined subset supports the interpretation that the detected hypointensities are iron-related.

As expected, the visibility of iron-related hypointensities on both T2w and iron-sensitive sequences improved with increasing magnetic field strength, reflecting fundamental susceptibility physics: higher magnetic fields amplify local magnetic field inhomogeneities induced by paramagnetic iron and, at higher spatial resolution, further enhance signal loss, resulting in greater lesion conspicuity. These effects have been well documented in human susceptibility-weighted imaging studies (69–71). These findings are also supported by Kimotsuki et al. (33), who observed T2w hypointensities consistent with iron accumulation in aging beagle dogs in an experimental setting using a pre-clinical 4.7 high-field scanner. Similarly, Aoki et al. (52) demonstrated early, region-specific hypointensities in the GP, red nucleus, and SN in young human individuals (aged 2–25 years) using a 1.5T.

### 4.3 Biological and translational implications

The complex role of iron in human neurobiology is increasingly recognized. In humans, it is well established that excessive iron accumulation contributes to oxidative stress through the Fenton reaction, generating reactive oxygen species (ROS) that promote mitochondrial dysfunction, lipid peroxidation, DNA damage, and ferroptosis, hallmarks of neurodegeneration (2, 5, 7, 9, 23, 41, 54, 72, 73). Therapeutic strategies in humans with Alzheimer’s disease, therefore, focus on strengthening neuronal antioxidant defenses by using glutathione peroxidase 4 (GPX4) and nuclear factor erythroid 2-related factor 2 (NRF2) as cellular protectors (29).

The GP and SN are particularly vulnerable due to their high metabolic demand. The GP and SN are already rich in iron under normal conditions, but are also among the first to show pathological iron accumulation in human neurodegenerative diseases (3). Given their roles in motor control and cognitive processing (55, 74), changes in these areas could also be clinically significant in companion animals (e.g., cognitive dysfunction syndrome). Similar regional susceptibility between the two brain regions was observed in our cohort. In our study, a direct comparison of iron visibility scores between the GP and SN was performed to assess for regional differences. Bowker’s test did not reveal any statistically significant differences in visibility distributions between these two brain regions across the whole dataset, as well as for the canine and feline subgroups.

Like humans, dogs undergo structural and cognitive brain changes with aging (75), including cortical atrophy and ventricular widening (31, 33), reduced size of the interthalamic adhesion (76), vascular abnormalities (e.g., cerebrovascular amyloid angiopathy), and accumulation of amyloid-beta plaques and tau phosphorylation (77, 78). Companion animals are increasingly recognized as valid translational models for human brain aging (26, 31, 72, 75, 79–81), offering the unique advantage that the time delay between MRI acquisition and neuropathologic validation can be controlled and minimized, facilitating correlation between imaging and tissue-based findings. Our findings strengthen the translational relevance of veterinary studies for human medicine.

Canine and feline cognitive dysfunction (dementia) remains an underrecognized and underdiagnosed neurodegenerative disorder in aging companion animals by owners and veterinarians. The prevalence is estimated at 14.2% in dogs older than 8 years (82), increasing to 67.3% in dogs older than 15 years (83). Typical symptoms include changes in activity, loss of spatial orientation, abnormal social interactions, disturbed sleep–wake cycles, house soiling, or increased vocalization (84, 85). Currently, there is no definitive diagnostic path, and the diagnosis is based on exclusion. Standard screening questionnaires, such as the Canine Dementia Scale (CADES), Canine Cognitive Assessment Scale (CCAS), and Canine Cognitive Dysfunction Rating Scale (CCDR), have been developed to aid in diagnosing cognitive dysfunction in dogs (84).

Importantly, recently published consensus guidelines for canine cognitive dysfunction (86) emphasize the increasing importance of advanced imaging in enhancing diagnostic confidence, disease staging, and distinguishing it from other age-related or structural brain disorders. Although none of the animals in our cohort had a confirmed diagnosis of cognitive dysfunction, the observed age-associated patterns of cerebral iron accumulation may reflect early or preclinical neurodegenerative changes. Owing to the predominantly retrospective study design, standardized longitudinal cognitive or behavioral assessments were unavailable; nevertheless, their inclusion would have enriched the dataset by providing meaningful clinical correlations.

The demonstrated association between cerebral iron accumulation and advancing age supports the potential value of MRI as a non-invasive imaging marker for early neurodegenerative processes. Early detection is crucial, as therapeutic interventions are most effective when initiated promptly (87–90). Future prospective studies incorporating cognitive and behavioral evaluations alongside imaging could help establish MRI as a valuable tool for diagnosing and monitoring age-related cerebral iron depositions in dogs and cats.

Some of the animals included in our study had a history of seizures, particularly idiopathic epilepsy. This is relevant considering findings by Martella et al. (44), who described a unilateral hypointense “pulvinar sign” on SWI as a marker of iron accumulation associated with seizure activity in human patients. Their results suggest that seizures may contribute to localized iron deposition through oxidative stress and disrupted iron metabolism. However, the bilateral hypointensities observed in our study are most consistent with physiologic or pathologic aging rather than focal seizure-related changes.

### 4.4 Limitations

Several limitations of this study should be acknowledged. Most notably, its retrospective design precluded standardized cognitive assessments and longitudinal follow-up, limiting the ability to determine clinical relevance or potential disease progression. Morphologic confirmation using iron-sensitive staining was unavailable in most cases because the animals were client-owned and typically presented with manageable conditions; staining was further limited to a subset of prospectively examined cases acquired on the 1.5T system using SWI. Image evaluation relied on a semi-quantitative visual grading system rather than on quantitative methods such as QSM or R2* mapping. While practical for clinical use, visual scoring is less precise and may be influenced by image quality; nonetheless, intraobserver agreement was good. The small number of cats (38 cats vs. 198 dogs) may have reduced statistical power for interspecies comparison. Finally, the use of different MRI scanners and iron-sensitive sequences may have introduced methodological heterogeneity, which is inherent to the retrospective clinical studies. However, all iron-sensitive sequences employed rely on the same susceptibility mechanism, and image evaluation was performed double-blinded, mitigating potential bias related to scanner or sequence differences.

## 5 Conclusion

This study provides the first large-scale veterinary evaluation of MRI changes consistent with cerebral iron deposition in the GP and SN of dogs and cats of various ages, using standard clinical T2w and iron-sensitive sequences (SWI, T2*) across commonly available high-field strengths. The results demonstrate that cerebral iron-associated hypointensities increase with age in both species and are more conspicuous on SWI or T2* than on conventional T2w imaging, supporting the potential role of iron-sensitive MRI as a non-invasive imaging marker of neuronal aging and possibly early neurodegenerative change. Improved detection of cerebral iron accumulation may allow earlier recognition of neurodegenerative processes, potentially preceding visible atrophy and providing added diagnostic value beyond standard T2w imaging; SWI or T2* should therefore be considered for inclusion in routine MRI protocols for older animals. Higher field strength (3T) further improves lesion conspicuity and diagnostic confidence.

Future prospective studies incorporating standardized cognitive testing, quantitative MRI techniques, and longitudinal follow-up are warranted to better assess the clinical significance of cerebral iron accumulation in aging companion animals.

## Data Availability

The raw data supporting the conclusions of this article will be made available by the authors, without undue reservation.
